# Radiological and functional lung sequelae of COVID-19: a systematic review and meta-analysis

**DOI:** 10.1186/s12890-021-01463-0

**Published:** 2021-03-22

**Authors:** Matsuo So, Hiroki Kabata, Koichi Fukunaga, Hisato Takagi, Toshiki Kuno

**Affiliations:** 1 Department of Medicine, Icahn School of Medicine at Mount Sinai, Mount Sinai Beth Israel, First Avenue, 16th Street, New York City, NY 10003 USA; 2 Division of Pulmonary Medicine, Department of Medicine, Keio University School of Medicine, Tokyo, Japan; 3 Division of Cardiovascular Surgery, Shizuoka Medical Center, Shizuoka, Japan

**Keywords:** Lung sequelae, COVID-19, Pulmonary function test, Computed tomography

## Abstract

**Background:**

The coronavirus disease 2019 (COVID-19) causes a wide spectrum of lung manifestations ranging from mild asymptomatic disease to severe respiratory failure. We aimed to clarify the characteristics of radiological and functional lung sequelae of COVID-19 patients described in follow-up period.

**Method:**

PubMed and EMBASE were searched on January 20th, 2021 to investigate characteristics of lung sequelae in COVID-19 patients. Chest computed tomography (CT) and pulmonary function test (PFT) data were collected and analyzed using one-group meta-analysis.

**Results:**

Our search identified 15 eligible studies with follow-up period in a range of 1–6 months. A total of 3066 discharged patients were included in these studies. Among them, 1232 and 1359 patients were evaluated by chest CT and PFT, respectively. The approximate follow-up timing on average was 90 days after either symptom onset or hospital discharge. The frequency of residual CT abnormalities after hospital discharge was 55.7% (95% confidential interval (CI) 41.2–70.1, *I*^2^ = 96.2%). The most frequent chest CT abnormality was ground glass opacity in 44.1% (95% CI 30.5–57.8, *I*^2^ = 96.2%), followed by parenchymal band or fibrous stripe in 33.9% (95% CI 18.4–49.4, *I*^2^ = 95.0%). The frequency of abnormal pulmonary function test was 44.3% (95% CI 32.2–56.4, *I*^2^ = 82.1%), and impaired diffusion capacity was the most frequently observed finding in 34.8% (95% CI 25.8–43.8, *I*^2^ = 91.5%). Restrictive and obstructive patterns were observed in 16.4% (95% CI 8.9–23.9, *I*^2^ = 89.8%) and 7.7% (95% CI 4.2–11.2, *I*^2^ = 62.0%), respectively.

**Conclusions:**

This systematic review suggested that about half of the patients with COVID-19 still had residual abnormalities on chest CT and PFT at about 3 months. Further studies with longer follow-up term are warranted.

**Supplementary Information:**

The online version contains supplementary material available at 10.1186/s12890-021-01463-0.

## Introduction

Coronavirus disease 2019 (COVID-19) is caused by a novel coronavirus known as severe acute respiratory syndrome coronavirus 2 (SARS-CoV-2) [1], which was identified to be the cause of pneumonia cases originated in Wuhan, a city in the providence of Hubei, China. COVID-19 infection rapidly spread to entire world, leading WHO to declare pandemic on March 11, 2020. As of February 24, 2021, WHO reported 111,593,583 cases and 2,475,020 deaths [2].

Although COVID-19 is known to cause multiple organ damages, pneumonia is the most frequent manifestation of infection ranging from mild asymptomatic cases to critical respiratory failure requiring ventilatory support [3]. Initial symptoms of COVID-19, lung complications, radiological features, and the management have been extensively reported. Importantly, persistent symptoms such as fatigue, dyspnea, joint pain, and chest pain in patients discharged from hospital at 60 days after symptom onset were reported [4]. During the worldwide outbreak of severe acute respiratory syndrome (SARS) in 2003, persistent residual lung fibrosis was reported in 62% of patients in chest computed tomography obtained on average 36.5 days after hospital admission [5] and can be still present in 7 years after symptom presentation [6]. In addition, impairment in diffusion capacity in SARS survivors has been reported in 25.5% of patients on average 40.5 days after hospital discharge [7, 8]. Another study also showed forced vital capacity < 80% predicted in 4.1% of patients and impaired diffusion capacity in 23.7% of patients at 1 year after disease onset [9, 10]. Similarly, studies of Middle East respiratory syndrome (MERS) survivors revealed that 33% of patients had chest radiograph abnormalities at 80 days after discharge [11] and 37% of patients had impaired diffusion capacity at 1 year after disease onset [12]. These radiological and functional lung sequelae can detrimentally affect survivors’ quality of life. Reports of lung sequelae regarding chest CT findings and PFT observed in patients with clinical recovery from COVID-19 has been increasing recently. Herein, we conducted this systematic review to clarify the characteristics of chest CT findings and PFT results in follow-up period after COVID-19.

## Method

### Protocol and registration

A review protocol does not exist for this analysis.

### Eligibility criteria

Included studies met the following criteria: the study design was an observational study that was published in peer-reviewed journals, the study population was patients with laboratory confirmed SARS-Cov-2 infections confirmed by using a quantitative real-time polymerase chain reaction (RT-PCR) who had follow-up evaluation of chest CT findings or PFT after recovery. Discharge criteria were either confirmed with two consecutive negative results of RT-PCR or clinical stability. Articles that do not contain original data of patients (e.g. guideline, editorial and review) or data obtained within 1 month of follow-up period after clinical recovery were excluded since the purpose of this review was to clarify the characteristics of lung sequelae in mid to long term follow-up period of patients with clinical recovery.

### Information sources and search

All observational studies which included patients with COVID-19 diagnosis and follow-up evaluation of chest CT findings or PFT after clinical recovery were identified using a 2-level strategy. Databases including PubMed and EMBASE were searched through January 20th, 2021. Search items included (SARS-CoV-2 or COVID-19 or COVID-19 [MH]) AND [follow-up OR long-term OR (long term)] AND ((Pulmonary function test or Respiratory function test [MH]) OR (computed tomography OR CT)).

### Study selection and data collection process

Relevant studies were identified through a manual search of secondary sources including references of initially identified articles, reviews, and commentaries. Two independent authors (M.S. and H.K.) reviewed the search results separately to select the studies based on inclusion and exclusion criteria. Disagreements were resolved by consensus.

### Data items

Outcomes included age, sex, comorbidities, initial COVID-19 symptoms, residual COVID-19 symptoms after hospital discharge, follow-up timing, disease severity, the proportion of abnormalities in chest CT, chest CT findings at follow-up and type of PFT abnormalities.

### Risk of bias in individual studies

Risk of bias in individual studies was reviewed using assessment of risk of bias in prevalence studies [13] (Additional file 1: Figures S2A, S2B).

### Summary measures and synthesis of results

To calculate frequency of residual lung abnormalities in follow up chest CT and PFT, retrospective and prospective studies focused on COVID-19 patients who had either follow up chest CT or PFT more than 1 month either after symptom onset or after discharge were utilized and the data regarding the proportion of CT abnormalities, their individual findings in chest CT, the frequency of total PFT abnormality, including obstructive lung function, restrictive lung function, and impaired diffusion capacity were combined using one-group meta-analysis in a random-effect model with DerSimonian-Laird method for continuous value and Wald method for discrete value with OpenMetaAnalyst version 12.11.14 (available from http://www.cebm.brown.edu/openmeta/). The frequency of comorbidities, initial COVID-19 symptoms, residual COVID-19 symptoms and proportion of severe cases were calculated by summation of events divided by the total number of patients from all studies the information is available. The clinical severity of COVID-19 was defined according to the WHO interim guidance [14] and the guidance from China “Pneumonia diagnosis and treatment program for novel coronavirus infection (trial version 5)” issued by National Health Commission of the People’s Republic of China [15] as follows; (1) mild disease: mild symptoms and no evidence of pneumonia in imaging, (2) moderate disease: fever, some respiratory infection symptoms and pneumonia on radiographic imaging, (3) severe disease: meet any of the followings, respiratory distress, respiratory rate > 30/min, SpO_2_ < 93% at rest, PaO_2_/FiO_2_ < 300 mmHg, (4) Critical disease: meet any of the followings, respiratory failure or requiring mechanical ventilation, shock or other organ failures requiring ICU monitoring. Publication bias was assessed by funnel plots with Egger’s test using Comprehensive Meta-Analysis version 3 (available from https://www.meta-analysis.com/index.php?cart=BTEJ5270189) [16].

## Results

### Study selection and study characteristics (Fig. 1)

**Fig. 1:**
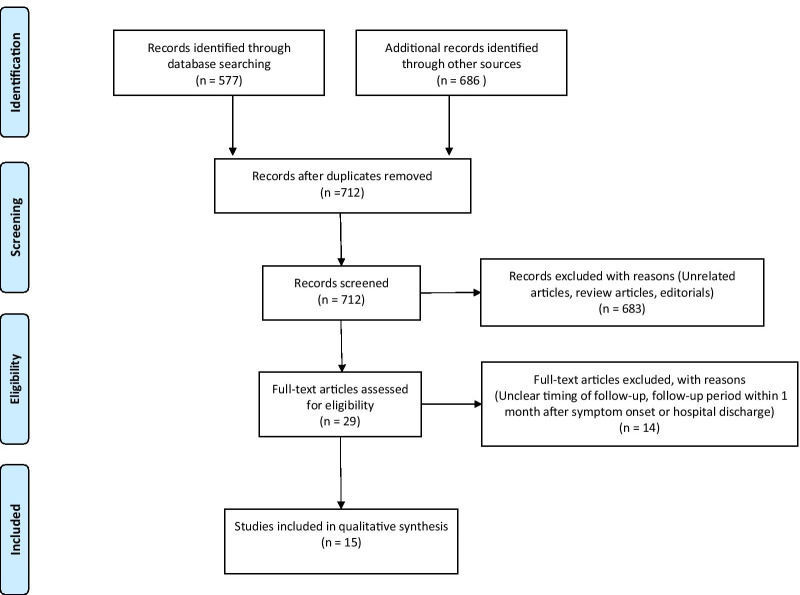
Flow diagram of study selection

We identified 712 articles in total that were reviewed based on the title and abstract. 683 articles were excluded as they were regarding irrelevant topics or did not contain original data. Among the 29 articles, 14 articles were excluded because either they did not clearly mention follow-up timing or the follow-up period was within 1 month after discharge or after symptom onset. Among 15 articles include data of baseline characteristics, 13 articles contained follow-up chest CT data and 10 articles contained follow-up PFT data. Clinical characteristics of extracted data are shown in Table 1. Among the 15 retrospective and prospective cohort studies with total of 3066 patients, 8 studies were from China and 7 studies were from other countries, including Iran, The Netherlands, Belgium, Canada, Norway, Italy, and Switzerland. They all clearly defined their population as COVID-19 patients who had either follow-up CT or pulmonary function tests more than 1 month after symptom onset or after discharge from hospital. The discharge criteria included two consecutive negative SARS-Cov2 nucleic acid tests detected at least 24 h apart each in 3 studies [17–19]. The decision to discharge was made clinically based on patients’ clinical status and per hospital policy in the other 12 studies [20–31]. Among these studies, 13 studies collected data of chest CT [17–25, 27, 29–31] and 10 studies collected data of PFT [17, 18, 20, 22, 24–29] from the patients with COVID-19 discharged during their study period. Risk of bias of each study is shown in Additional file 1: figure S2.

**Table 1:** Results of systematic review with cohort studies of COVID-19 patients who had follow-up—baseline characteristics

Author	Country	Publication date	Study design	Cohort Size, N	Follow-up timing	Age	Male, % (N)	Comorbidities, % (N)	Initial COVID-19 Symptoms, % (N)	Symptoms at follow-up, % (N)	Severe cases, % (N)	Non-severe cases, % (N)
You [17]	China	6/5/20	Retrospective	18	38 ± 13.4 days after discharge	50.7 ± 12.1	55.6% (10)	Hypertension 16.7% (3) Diabetes mellitus 5.6% (1) Hypothyroidism 5.6% (1)	NA	NA	33.3% (6)	66.7% (12)
Huang [18]	China	6/29/20	Retrospective	57	30 days after discharge	46.7 ± 13.8	45.6% (26)	Hypertension 19.3% (11) Diabetes mellitus 7.0% (4) Cardiovascular disease 5.3% (3) Malignancy 5.3% (3)	NA	Cough 10.5% (6) Dyspnea 7% (4) Occasional wheezing 5.3% (3)	29.8% (17)	70.2% (40)
Liu [19]	China	7/21/20	Retrospective	51	Initial CT: median 10 days (range 7–16) after discharge Llatest CT: median 31 days (range 20–37) after initial CT	46.6 ± 13.9	41.2% (21)	Hypertension 13.7% (7) Diabetes mellitus 7.8% (4) Coronary heart disease 2% (1)	NA	Cough 15.7% (8) Sputum 3.9% (2) Throat discomfort 5.9% (3)	NA	NA
Zhao [20]	China	8/25/20	Retrospective	55	64–93 days after discharge	47.7 ± 15.5	58.2% (32)	Hypertension 10.9% (6) Diabetes mellitus 3.6% (2) Cardiovascular disease 3.6% (2)	Fever 67.3% (37) Cough 54.5% (30) Fatigue 32.7% (18)	GI symptoms 30.9% (17) Headache 18.2% (10) Fatigue 16.4% (9) Exertional dyspnea 14.6% (8) Cough and sputum 1.8% (1)	7.3% (4)	92.7% (51)
Zhong [21]	China	10/14/20	Retrospective	52	39.6 ± 5.96 days after symptom onset 19.71 ± 4.08 days after discharge	45.5 ± 13.7	55.8% (29)	Hypertension 23.1% (12) Diabetes mellitus 9.6% (5) Cardiac disease 5.8% (3) Cerebrovascular disease 1.9% (1)	Fever 100% (52) Cough 48.1% (25) Fatigue 28.8% (15) Headache 7.7% (4) Vomiting 1.9% (1) Abdominal pain3.8% (2) Diarrhea 1.9% (1)	NA	36.5% (19)	63.5% (33)
Liang [22]	China	10/26/20	Prospective	76	3 months after discharge	41.3 ± 13.8	28% (21)	Hypertension 6.6% (5) Cardiovascular disease 1.3% (1) Diabetes mellitus 3.9% (3) Thyroid disease 2.6% (2) Pulmonary tuberculosis 6.6% (5) Chronic bronchitis 3.9% (3) Asthma 2.6% (2)	NA	NA	9.2% (7)	90.8% (69)
Tabatabaei [23]	Iran	11/9/20	Retrospective	52	91 ± 15.5 days after initial CT	50.2 ± 13.1	61.5% (32)	Cardiac disease 11.5% (6) Diabetes mellitus 7.7% (4) Hypertension 3.8% (2) Pulmonary disease 3.8% (2)	Fever 88.4% (46) Fatigue 53.8% (28) Dyspnea 40.4% (21)	Mild chest pain or discomfort 28.8% (15) Dyspnea on exertion 11.5% (6) Cough 1.9% (1)	NA	NA
van den Borst [24]	The Netherlands	11/21/20	Prospective	124	13.0 ± 2.2 weeks after symptom onset 9.1 ± 1.6 weeks after discharge	59 ± 14	60% (74)	Cardiovascular disease 24% (30) Asthma 10% (12) COPD 6% (7) Hypertension 28% (34) Diabetes mellitus 14% (17) Chronic kidney disease 8% (10) Malignancy 20% (25) Immunocompromised status 15% (18)	NA	NA	37.1% (46)	62.9% (78)
Smet [25]	Belgium	11/30/20	Retrospective	220	74 ± 12 days after diagnosis	53 ± 13	61.4% (135)	Hypertension 34.1% (75) Diabetes mellitus 17.7% (39)	NA	Fatigue 40.9% (90) Dyspnea 29.5% (65)	100% (220)	0% (0)
Shah [26]	Canada	12/3/20	Prospective	60	12 weeks after symptom onset	67 [54, 74]	68.3% (41)	Hypertension 35% (21) Diabetes 22% (13) Chronic pulmonary disease 13% (8) Coronary artery disease 10% (6) Malignancy 10% (6) Chronic Kidney Disease 7% (4)	NA	Dyspnea 20% (12) Cough 20% (12)	76.7% (46)	23.3% (14)
Lerum [27]	Norway	12/10/20	Prospective	103	83 [73, 90] days after hospital admission	59 [49, 72]	52% (54)	Hypertension 34.0% (35) Diabetes Mellitus 7.8% (8)	NA	NA	14.6% (15)	85.4% (88)
Bellan [28]	Italy	1/4/21	Prospective	238	4 months after after discharge	61 [50, 71]	59.7% (142)	Hypertension 41.2% (98) Diabetes Mellitus 15.1% (36) Dyslipidemia 8.4% (20) COPD 5.8% (14) Inflammatory bowel disease 1.7% (4) Chronic liver disease 2.9% (7) Autoimmune disease 2.1% (5) Hematological disease 6.3% (15) Chronic kidney disease 6.3% (15)	Fever 90.3% (215) Cough 55.5% (132) Dyspnea 54.2% (129) Ageusia 29.4% (70) Anosmia 26.5% (63) Diarrhea 22.7% (54) Arthralgia 19.3% (46) Myalgia 18.9% (45) Chest pain 0.8% (2) Sore throat 0.4% (1) Headache 0.4% (1)	Cough 2.5% (6) Dyspnea 5.5% (13) Ageusia 5.0% (12) Anosmia 4.6% (11) Diarrhea 1.3% (3) Arthralgia 5.9% (14) Myalgia 5.9% (14) Chest pain 0.4% (1)	29.4% (70)	70.6% (168)
Huang [29]	China	1/8/21	Ambi-directional	1733	186 [175, 199] days after symptom onset	57 (47, 65)	52% (897)	Hypertension 29.1% (505) Diabetes Mellitus 11.9% (207) Cardiovascular disease 7.4% (128) Cerebrovascular disease 2.7% (47) Malignancy 2.5% (44) COPD 1.8% (31) Chronic Kidney Disease 1.6% (27)	NA	Fatigue 62.7% (1038) Sleep difficulties 26.4% (437) Hair loss 21.7% (359) Smell disorder 10.6% (176) Palpitations 9.3% (165) Arthralgia 9.3% (165) Decreased appetite 8.3% (138) Taste disorder 7.3% (120) Dizziness 6.1% (101) Diarrhea or vomiting 4.8% (80) Chest pain 4.5% (75) Sore throat 4.2% (69) Skin rash 2.8% (47) Myalgia 2.4% (39) Headache 2.0% (33)	7.0% (122)	93.0% (1611)
Guler [30]	Switzerland	1/8/21	Prospective	113	128 [108, 144] days after symptom onset	57.2 ± 12.1	59.3% (67)	Hypertension 35.4% (40) Diabetes Mellitus 20.4% (23) Interstitial lung disease 4.4% (5) COPD 8.0% (9) Asthma 13.3% (15) GERD 9.7% (11) Sleep apnea 10.6% (12) Chronic heart failure 9.7% (11) Chronic Kidney Disease 11.5% (13) Malignancy 5.3% (6)	NA	NA	58.4% (66)	41.6% (47)
Han [31]	China	1/26/21	Prospective	114	175 ± 20 days after symptom onset	54 ± 12	70.2% (80)	Hypertension 28.1% (32) Diabetes Mellitus 11.4% (13) Chronic pulmonary disease 14.0% (16)	NA	Cough 6.1% (7) Sputum 10.0% (11) Exertional Dyspnea 14.0% (16)	NA	NA

Value is shown as mean ± SD or median [Q1, Q3]. Abbreviations: *NA* non-applicable, *PFT* pulmonary function test, *CT* computed tomography, *GI* gastrointestinal. Studies are in order of publication date

### Baseline characteristics of individual studies (Table 1)

The follow-up timing of chest CT or pulmonary function tests varied from 1 to 6 months after symptom onset. The average approximate follow-up timing after either symptom onset or hospital discharge was 90 days. Mean age was 56.0 ± 14.3, and 54.2% of the cohort was male. Baseline comorbidities were reported in 15 studies: hypertension 28.9% (886/3066), diabetes mellitus 12.4% (379/3066), cardiovascular disease 6.2% (191/3066), chronic pulmonary disease including asthma, chronic obstructive pulmonary disease, chronic bronchitis, pulmonary tuberculosis and interstitial lung disease 3.6% (110/3066), malignancy 2.7% (84/3066), chronic kidney disease 2.3% (69/3066) and cerebrovascular disease 1.6% (48/3066). Initial COVID-19 symptoms were reported in 4 studies: fever 88.2% (350/397), cough 47.1% (187/397), dyspnea 37.8% (150/397), ageusia 17.6% (70), anosmia 15.9% (63/397), fatigue 15.4% (61/397), diarrhea 13.9% (55/397), arthralgia 11.6% (46/397), myalgia 11.3% (45/397). Residual symptoms at follow-up were reported in 9 studies: fatigue 44.1% (1137/2580), sleep difficulty 16.9% (437/2580), hair loss 13.9% (359/2580), anosmia 7.2% (187/2,580), arthralgia 6.9% (179/2580), palpitation 6.4% (165/2580), decreased appetite 5.3% (138/2580), ageusia 5.1% (132/2580), and dyspnea 4.3% (112/2580). Severe COVID-19 diseases were observed in 22.4% (638/2849), and mild to moderate cases were observed in 77.6% (2211/2849).

### Follow-up CT results after discharge (Table 2)

**Table 2:** Results of systematic review with cohort studies of COVID-19 patients—chest CT

Author	Follow-up timing of latest tests	Cohort Size, N	Residual CT abnormalities % (N)	Parenchymal band or fibrous stripe % (N)	GGO % (N)	Consolidation % (N)	Interstitial thickening or interlobular septal thickening % (N)	Bronchovascular bundle distortion or bronchiectasis % (N)	Thickening of adjacent pleura % (N)	Pleural effusion % (N)	Crazy paving % (N)
You [17]	PFT Non-severe cases: 40 ± 11.6 days Severe cases: 34.7 ± 16.5 days CT was taken closest to the date of PFT	18	83% (15)	NA	61.1% (11)	NA	NA	NA	NA	NA	NA
Huang [18]	30 days after discharge	57	54.4% (31)	NA	NA	NA	NA	NA	NA	NA	NA
Liu [19]	31 (20–37) days after discharge	51	35.3% (18)	NA	33.3% (17)	2.0% (1)	35.3% (18)	3.9% (2)	23.5% (12)	NA	NA
Zhao [20]	64–93 days after discharge	55	70.9% (39)	NA	12.7% (7)	NA	27.3% (15)	NA	41.8% (23)	1.8% (1)	5.5% (3)
Zhong [21]	39.6 ± 6.0 days after symptom onset 19.7 ± 4.1 days after discharge	52	69.2% (36)	36.5% (19)	63.5% (33)	9.6% (5)	5.8% (3)	7.7% (4)	NA	NA	NA
Liang [22]	3 months after discharge	21	23.8% (5)	NA	23.8% (5)	NA	NA	NA	NA	NA	NA
Tabatabaei [23]	91 ± 15.5 days after initial CT	52	42.3% (22)	19.2% (10)	36.5% (19)	NA	NA	NA	NA	NA	NA
van den Borst [24]	13.0 ± 2.2 weeks after symptom onset 9.1 ± 1.6 weeks after discharge	84	90.5% (76)	64.3% (54)	86.9% (73)	NA	NA	60.7% (51)	NA	NA	NA
Smet [25]	74 ± 12 days after diagnosis	220	26.4% (58)	NA	25.0% (55)	3.6% (8)	NA	NA	NA	NA	40.9% (90)
Lerum [27]	83 [73, 90] days after hospital admission	103	NA	18.4% (19)	23.3% (24)	NA	NA	NA	NA	NA	NA
Huang [29]	186 [175, 199] days after symptom onset	353	52.7% (186)	15.9% (56)	44.8% (158)	1.1% (4)	0.8% (3)	NA	4.2% (15)	NA	NA
Guler [30]	128 [108, 144] days after symptom onset	52	NA	51.9% (27)	57.7%(30)	28.8% (15)	1.9% (1)	38.5% (20)	1.9% (1)	NA	NA
Han [31]	175 ± 20 days after symptom onset	114	62.3% (71)	NA	62.3% (71)	23.7% (27)	NA	10.5% (12)	35.1% (40)	8.8% (10)	NA

Value is shown as mean ± SD or median [Q1, Q3]. Abbreviations: *CT* computed tomography, *GGO* ground glass appearance, *NA* non-applicable, *PFT* pulmonary function test. Studies are in order of publication date.

**Fig. 2:**
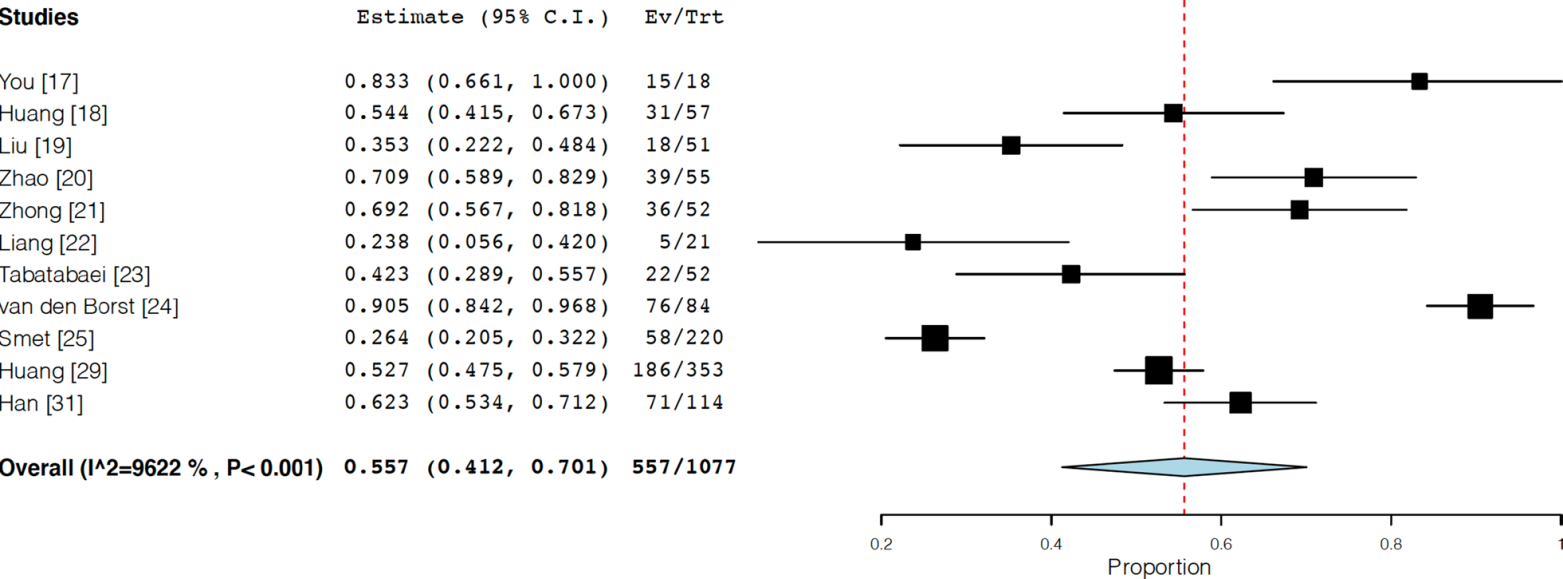
Forest plots for follow-up chest CT results (random-effects model); frequency of CT abnormalities observed after hospital discharge

13 studies were eligible to assess the residual chest CT findings [17–25, 27, 29–31]. The average approximate follow-up timing after either symptom onset or hospital discharge was 90 days. The frequency of CT abnormalities observed was 55.7% (95% confidential interval (CI) 41.2–70.1, *I*^2^ = 96.2%) (Fig. 2). The proportion of each finding observed was as follows; ground glass opacity: 44.1% (95% CI 30.5–57.8, *I*^2^ = 96.2%), parenchymal band or fibrous stripe: 33.9% (95% CI 18.4–49.4, *I*^2^ = 95.0%), thickening of adjacent pleura: 19.9% (95% CI 8.7–31.1, *I*^2^ = 95.4%), bronchovascular distortion or bronchiectasis: 23.7% (95% CI 6.4–40.9, *I*^2^ = 96.3%), interstitial thickening or interlobular septal thickening: 11.1% (95% CI 3.7–18.4, *I*^2^ = 91.6%), consolidation: 8.8% (95% CI 3.9–13.8, *I*^2^ = 91.0%), pleural effusion: 5.0% (95% CI − 1.8–11.8, *I*^2^ = 78.8%) (Additional file 1: Figure S1A–S1G).

### Follow-up pulmonary function test after discharge (Table 3)

**Table 3:** Results of systematic review with cohort studies of COVID-19 patients—pulmonary function test

Author	Follow-up timing after discharge	Cohort Size, N	PFT abnormalities at follow-up, % (N)	Restrictive pattern % (N) VCmax % predicted < 80% or VCmax < LLN OR FVC % predicted < 80% or FVC < LLN OR TLC z-score < − 1.64 or TLC % predicted < 80%	Obstructive pattern % (N) FEV1/FVC < 70% OR FEV1/VCmax < LLN %	Diffusion Impairment % (N) DLCO < 80% predicted OR DLCO < LLN
You [17]	38 ± 13.4 days after discharge	18	38.9% (7)	16.7% (3)	16.7% (3)	NA
Huang [18]	30 days after discharge	57	NA	10.5% (6)	1.8% (1)	52.6% (30)
Zhao [20]	64–93 days after discharge	55	25.5% (14)	10.9% (6)	9.1% (5)	16.4% (9)
Liang [22]	3 months after discharge	76	42.1% (32)	NA	6.6% (5)	19.7% (15)
van den Borst [24]	13.0 ± 2.2 weeks after symptom onset 9.1 ± 1.6 weeks after discharge	84	NA	9.5% (8)	15.5% (13)	48.8% (41)
Smet [25]	74 ± 12 days after diagnosis	220	54.1% (119)	38.2% (84)	NA	21.8% (48)
Shah [26]	12 weeks after symptom onset	60	58.3% (35)	23.3% (14)	11.7% (7)	51.7% (31)
Lerum [27]	83 [73, 90] days after hospital admission	1032	NA	6.8% (7)	NA	23.3% (24)
Bellan [28]	4 months after after discharge	224	NA	NA	NA	50.4% (113)
Huang [29]	186 [175, 199] days after symptom onset	349	NA	16.0% (56)	6.3% (22)	32.7% (114)

Value is shown as mean ± SD or median [Q1, Q3]. Abbreviations: *PFT* pulmonary function test, *LLN* lower limit of normal, *VC* vital capacity, *FVC* forced vital capacity, *TLC* total lung capacity, *FEV1* forced expiratory volume in 1 s, *D*_*LCO*_ diffusion capacity of the lungs for carbon, *NA* non-applicable. Studies are in order of publication date

**Fig. 3:**
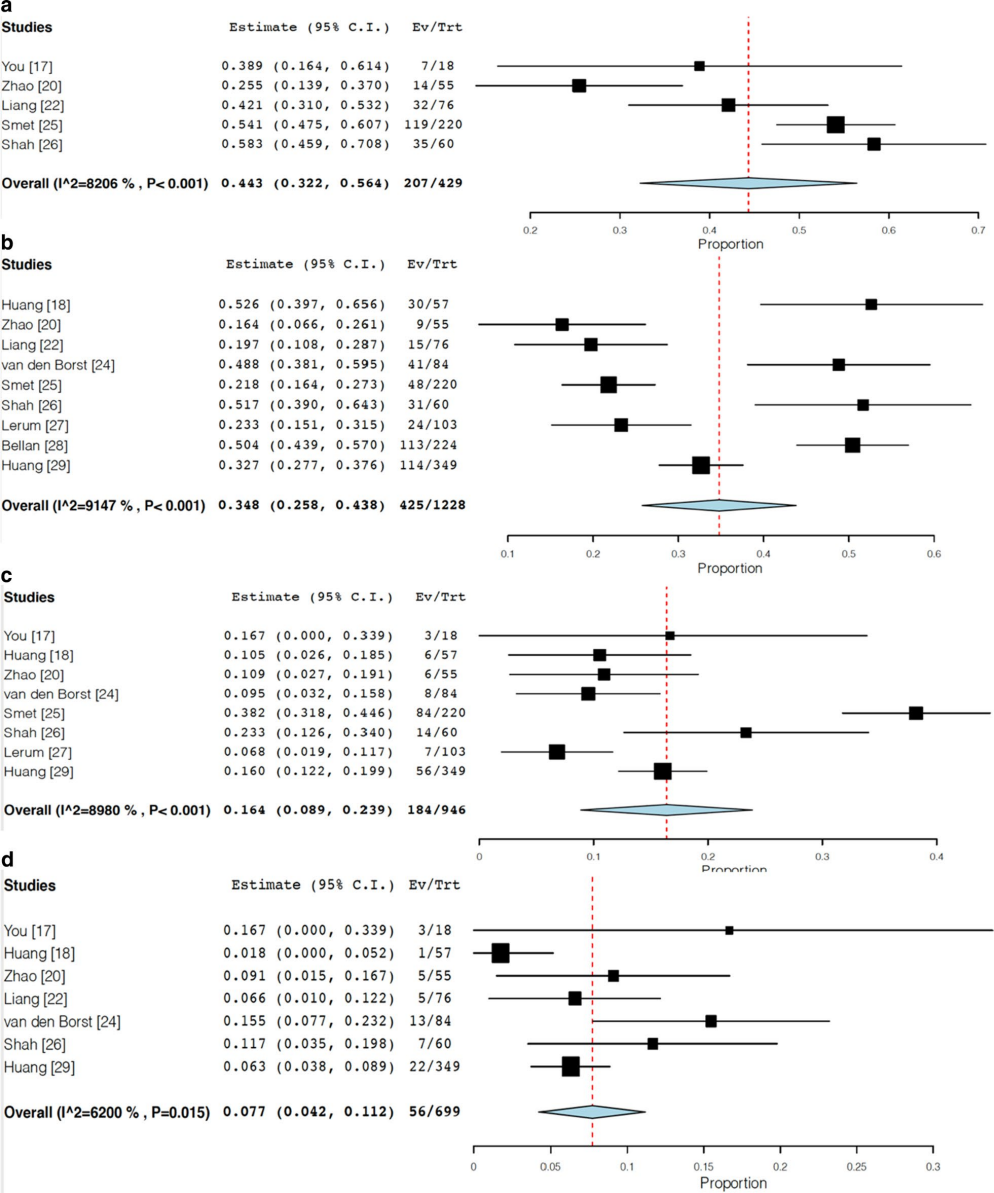
Forest plots for follow-up PFT results (random-effects model). **a** Frequency of PFT abnormalities observed after hospital discharge, **b** frequency of impaired diffusion capacity in follow-up PFT, **c** frequency of restrictive pattern in follow-up PFT, **d**: frequency of obstructive pattern in follow-up PFT

We identified 10 studies regarding PFT results in follow up period after 1 month [17, 18, 20, 22, 24–29]. The follow-up timing was approximately 90 days on average. The frequency of follow-up pulmonary function test abnormalities was 44.3% (95% CI 32.2–56.4, *I*^2^ = 82.1%) (Fig. 3A). Impaired diffusion capacity was observed in 34.8% of patients (95% CI 25.8–43.8, *I*^2^ = 91.5%) (Fig. 3B). Restrictive pattern and obstructive pattern were observed in 16.4% (95% CI 8.9–23.9, *I*^2^ = 89.8%) (Fig. 3C) and 7.7% (95% CI 4.2–11.2, *I*^2^ = 62.0%) of patients (Fig. 3D).

## Discussion

The salient findings of our systematic review are the following; (1) the frequency of CT abnormalities after hospital discharge was 55.7% (95% CI 41.2–70.1),Ground glass opacity and parenchymal bands/fibrous stripe were the most frequent findings; (2) the frequency of PFT abnormalities after hospital discharge was 44.3% (95% CI 32.2–56.4). Despite relatively low frequency of restrictive or obstructive pulmonary dysfunction, impaired diffusion capacity was the most prominent findings among these PFT results. It is noteworthy that the frequency of chest CT abnormalities was high despite the high proportion of non-severe cases (77.6%, 2211/2849 patients) in this combined data. As previously described in studies from outbreaks of SARS [5], our combined data regarding the frequency of chest CT abnormalities observed in follow-up period of about 3 months in COVID-19 patients was about 60%, and the most frequently observed functional lung sequelae was impaired diffusion capacity. Our combined data of decreased diffusion capacity frequency was higher than that reported in SARS in a similar follow-up period [7, 8]. Furthermore, compared to radiological lung sequelae of MERS, our data revealed higher rate of residual CT abnormalities [11].

Interestingly, despite the absence of macro level of lung dysfunction represented as reduced lung volume (restrictive lung dysfunction) or impaired airway dynamics (obstructive lung dysfunction), impaired diffusion capacity was more prominent, which indicates the disorder of interstitial structure and microvasculature of lungs. This result may represent underlying microthrombus formation in the lungs as previously reported in autopsy cases of COVID-19 diseases [32–35]. Hypercoagulable state in COVID-19 has been reported more and more frequently [36, 37], leading to the robust use of inpatient thromboprophylaxis and extended thromboprophylaxis following hospital discharge for select patients [38]. As demonstrated by Zhao et al. [20], elevated serum D-dimer was associated with decreased diffusion capacity in follow-up PFT. This finding is also consistent with possible microthrombus formation as underlying pathophysiology of COVID-19 disease. Finally, British Thoracic Society guidance on Respiratory follow up of patients with a clinic-radiological diagnosis of COVID-19 pneumonia has defined follow-up algorithms for COVID-19 pneumonia patients, which suggests to obtain chest radiography follow-up at 12 weeks after discharge and consider full PFT based on severity of COVID-19 disease. Any abnormalities in these tests encourage us to take high resolution CT or CT pulmonary angiography for possible residual interstitial lung disease or pulmonary embolism and recommend referral to either interstitial lung disease or pulmonary hypertension specialist services [39].

This study has several limitations. First, the description of follow up timing was variable and inconsistent between studies such as different starting point of duration and scale of duration, which made it difficult to precisely compare the proportion of patients with residual abnormalities. Second, each article reported CT abnormalities findings with different radiological terminology and PFT abnormalities were reported with different definition of restrictive or obstructive pattern, which made it difficult to accurately assess the proportion of each finding. Third, some studies reported only moderate severity of COVID-19 cases while others included moderate to critical diseases, which can be a factor leads to selection bias as well as low participation of patients in some studies which leads to non-response bias (Additional file 1: Figure S2A–S2B). More follow up data need to be published in the near future and further depict the long-term characteristics of radiological findings and lung function in COVID-19 disease.

## Conclusion

This systematic review assessed the post discharge chest CT and pulmonary function tests in COVID-19 patients in follow-up period of about 3 months. The frequency of residual chest CT abnormalities observed was 55.7%, and ground glass opacity and parenchymal band were most frequent. Follow-up pulmonary function test was abnormal in 44.3%, mainly presenting decreased diffusion capacity. Further studies with longer term follow-up data are warranted to clarify how long these abnormalities are persistent, which will be helpful to manage patients with long-term sequelae from COVID-19 disease.

## Supplementary Information

**Additional file 1: Supplemental Figure S1**. Forrest plots of the proportion of (A) ground glass opacity in follow-up chest CT; (B) parenchymal band or fibrous stripe in follow-up chest CT; (C) adjacent pleural thickening in follow-up chest CT; (D) bronchovascular distortion or bronchiectasis in follow-up chest CT; (E) interstitial thickening or interlobular septal thickening in follow-up chest CT; (F) consolidation in follow-up chest CT; (G) pleural effusion in follow-up chest CT. **Supplemental Figure S2**. Risk of bias (A): Risk of bias graph: review authors’ judgements about each risk of bias item presented as percentages across all included studies (B): Risk of bias summary: review authors’ judgements about each risk of bias item for each included study. +, low risk of bias; –, high risk of bias. **Supplemental Figure S3**. Publication bias Funnel plots of precision by point estimate of (A) chest CT abnormalities at follow-up; (B) PFT abnormalities at follow-up; (C) impaired DLCO at follow-up; (D) restrictive pattern in follow-up PFT; (E) obstructive pattern in follow-up PFT.

## Data Availability

We used the data from published data given its nature of systematic review and meta-analysis.
