# Supplementary material for: Radiological and functional lung sequelae of COVID-19: a systematic review and meta-analysis
Source: BMC Pulm Med. 2021 Mar 22;21:97. doi: 10.1186/s12890-021-01463-0 (PMC7983097; doi:10.1186/s12890-021-01463-0)
Supplement: Supplementary file 1 — Additional file 1: Supplemental Figure S1. Forrest plots of the proportion of (A) ground glass opacity in follow-up chest CT; (B) parenchymal band or fibrous stripe in follow-up chest CT; (C) adjacent pleural thickening in follow-up chest CT; (D) bronchovascular distortion or bronchiectasis in follow-up chest CT; (E) interstitial thickening or interlobular septal thickening in follow-up chest CT; (F) consolidation in follow-up chest CT; (G) pleural effusion in follow-up chest CT. Supplemental Figure S2. Risk of bias (A): Risk of bias graph: review authors’ judgements about each risk of bias item presented as percentages across all included studies (B): Risk of bias summary: review authors’ judgements about each risk of bias item for each included study. +, low risk of bias; –, high risk of bias. Supplemental Figure S3. Publication bias Funnel plots of precision by point estimate of (A) chest CT abnormalities at follow-up; (B) PFT abnormalities at follow-up; (C) impaired DLCO at follow-up; (D) restrictive pattern in follow-up PFT; (E) obstructive pattern in follow-up PFT. [file 12890_2021_1463_MOESM1_ESM.docx]

**Radiological and Functional Lung Sequelae of COVID-19: Systematic Review and meta-analysis**

Matsuo So, MD^a^, Hiroki Kabata, MD, PhD^b^, Koichi Fukunaga, MD, PhD^b^, Hisato Takagi, MD, PhD^c^, Toshiki Kuno, MD^a,^ PhD

^a^ Department of Medicine, Icahn School of Medicine at Mount Sinai, Mount Sinai Beth Israel, New York City, New York, USA.

^b^ Division of pulmonary medicine, Department of Medicine, Keio University School of Medicine, Tokyo, Japan

^c^ Division of Cardiovascular Surgery, Shizuoka Medical Center, Shizuoka, Japan

**Supplemental Figure Legends**

Supplemental Figure S1: Forrest plots of the proportion of (A) ground glass opacity in follow-up chest CT; (B) parenchymal band or fibrous stripe in follow-up chest CT; (C) adjacent pleural thickening in follow-up chest CT; (D) bronchovascular distortion or bronchiectasis in follow-up chest CT; (E) interstitial thickening or interlobular septal thickening in follow-up chest CT; (F) consolidation in follow-up chest CT; (G) pleural effusion in follow-up chest CT

Supplemental Figure S2: Risk of bias

(A): Risk of bias graph: review authors’ judgements about each risk of bias item presented as percentages across all included studies

(B): Risk of bias summary: review authors’ judgements about each risk of bias item for each included study. +, low risk of bias; –, high risk of bias

Supplemental Figure S3: Publication bias

Funnel plots of precision by point estimate of (A) chest CT abnormalities at follow-up; (B) PFT abnormalities at follow-up; (C) impaired DLCO at follow-up; (D) restrictive pattern in follow-up PFT; (E) obstructive pattern in follow-up PFT

Supplemental Figure S1A


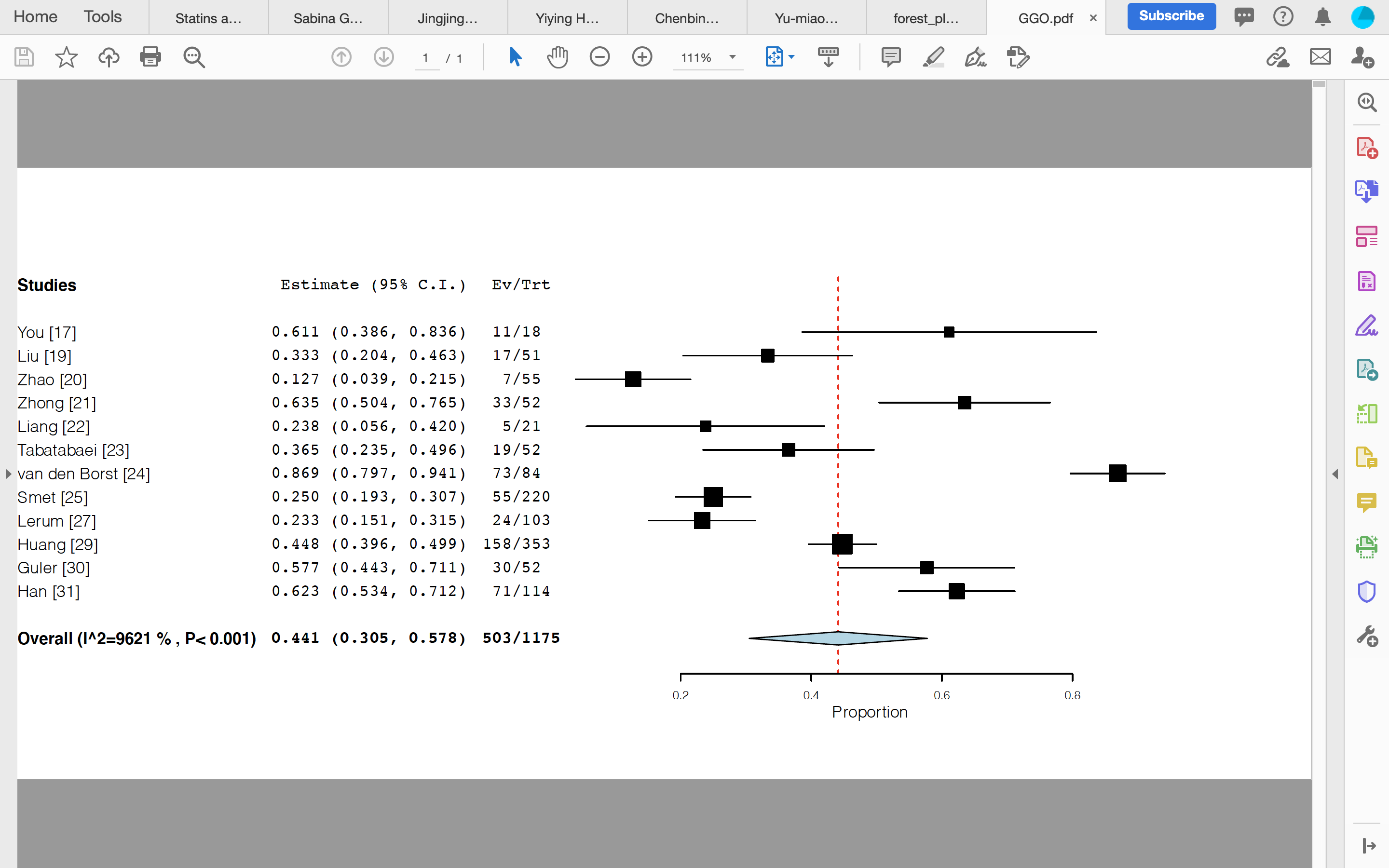


Supplemental Figure S1B


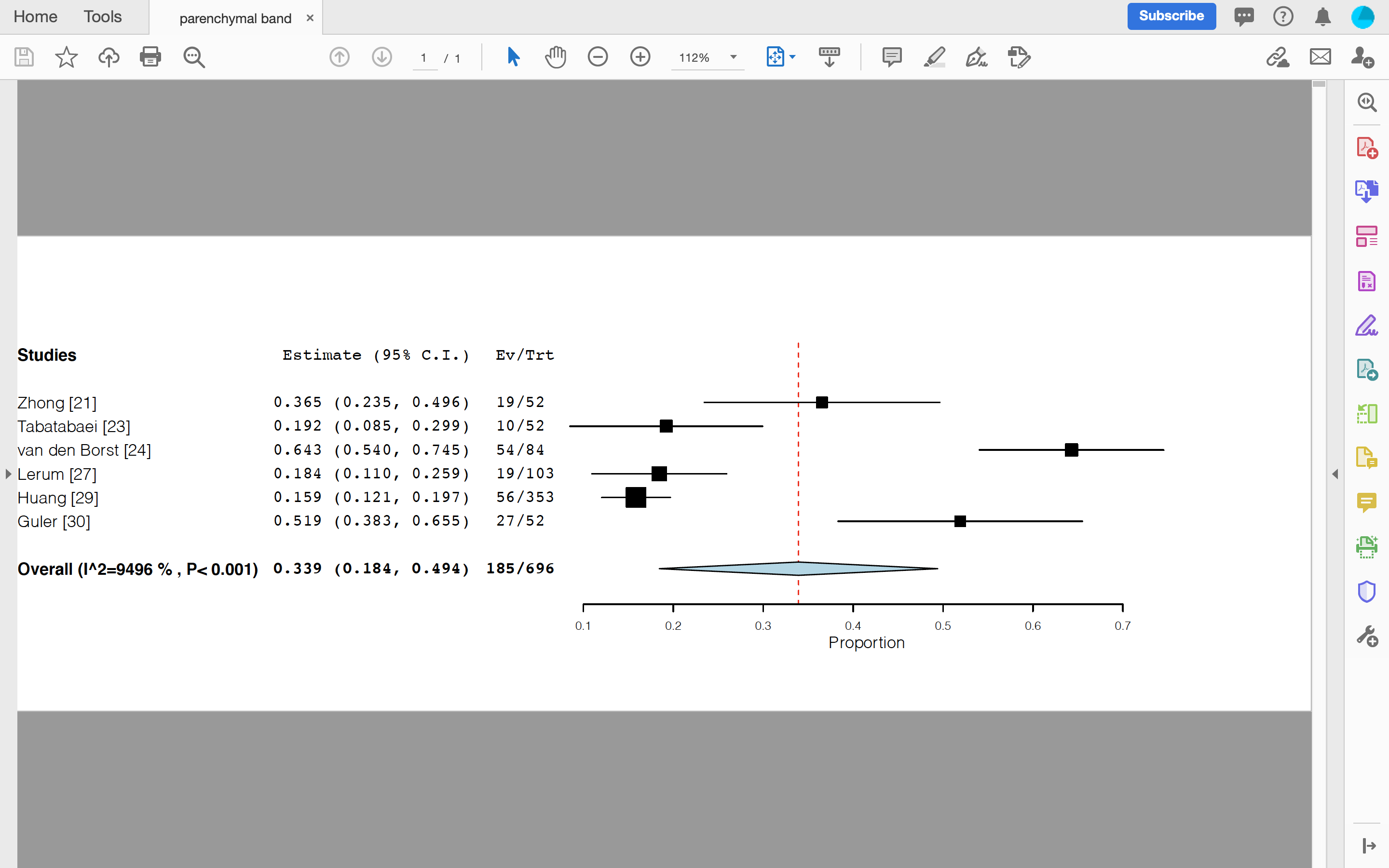


Supplemental Figure S1C


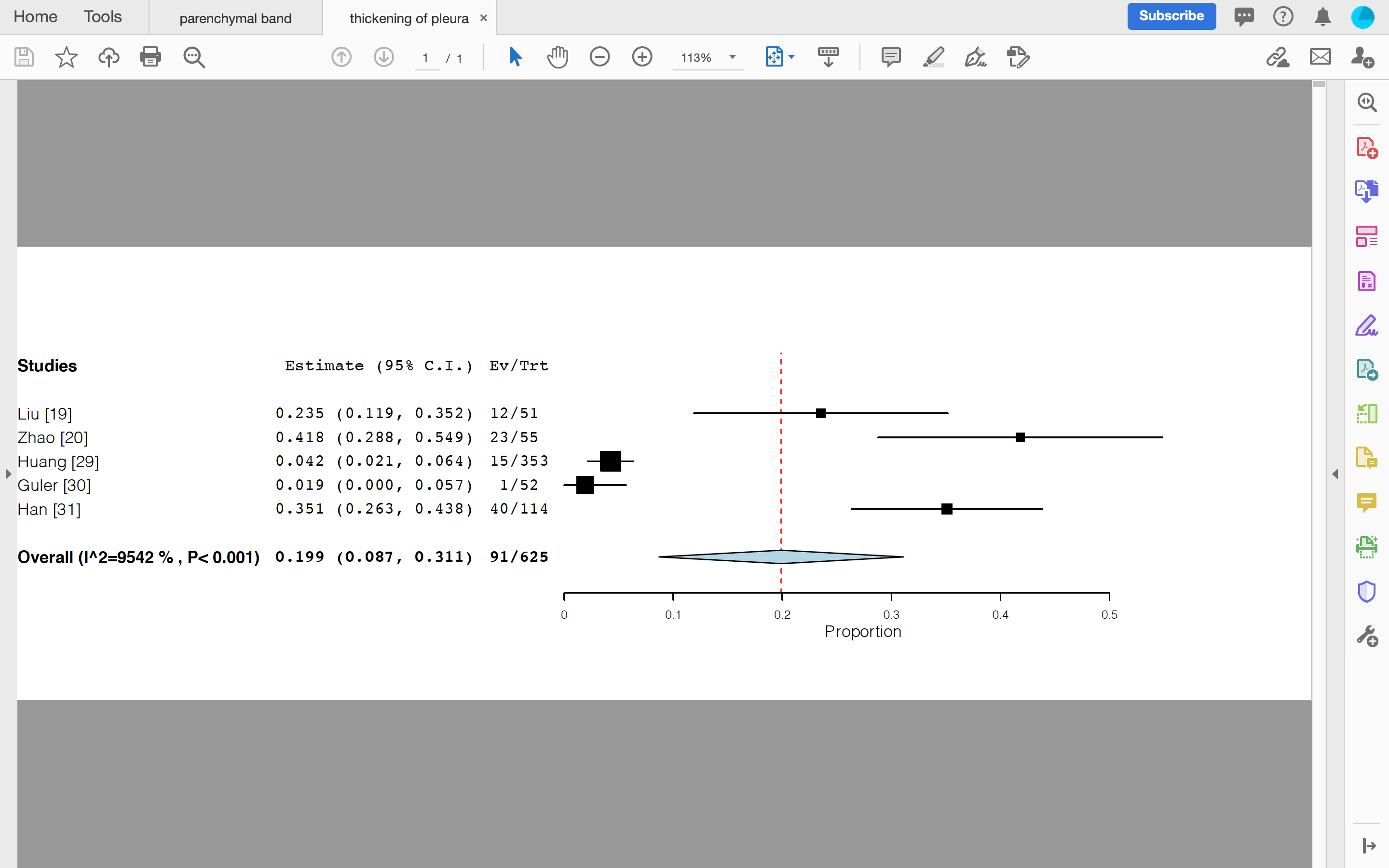


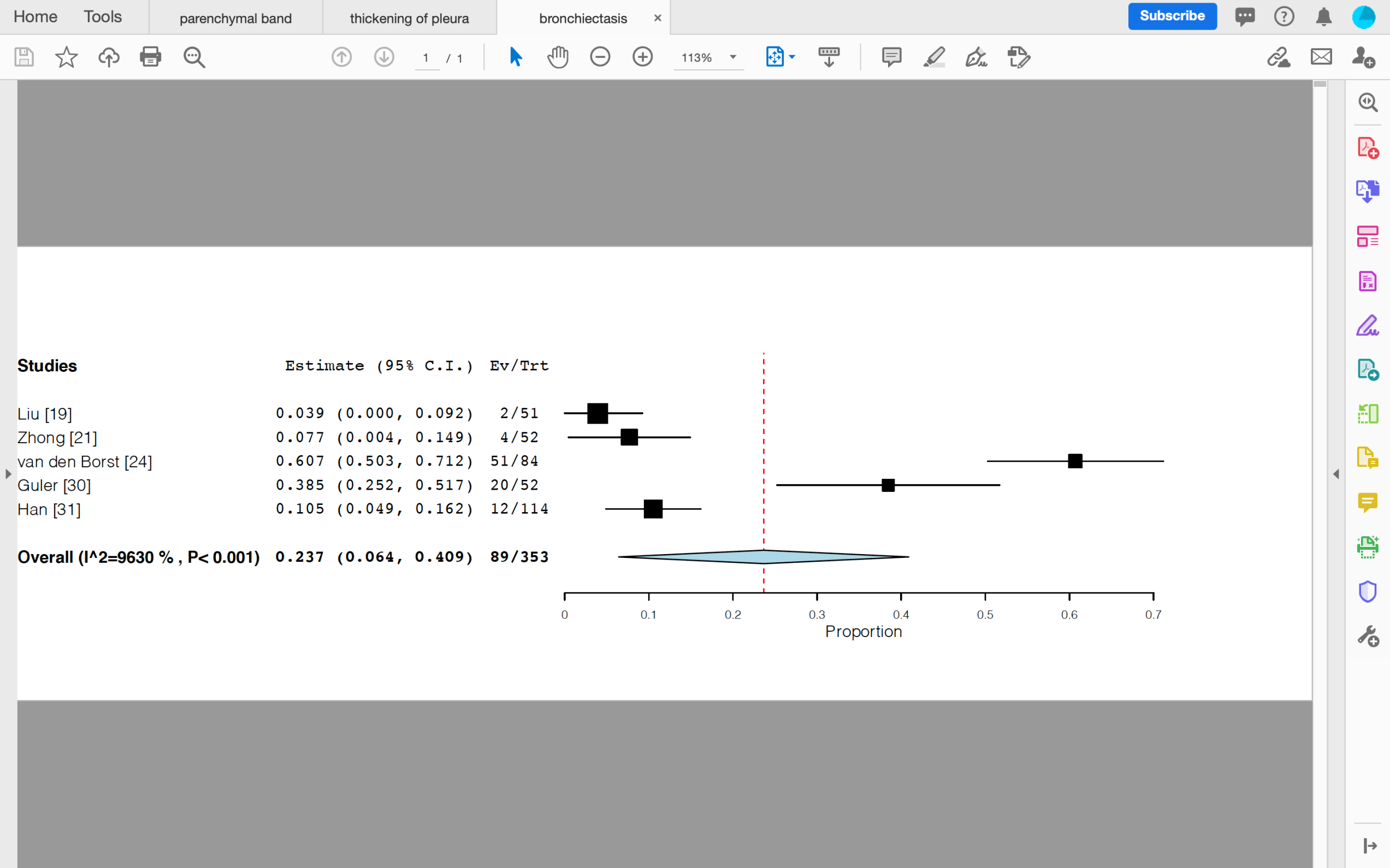
Supplemental Figure S1D

Supplemental Figure S1E


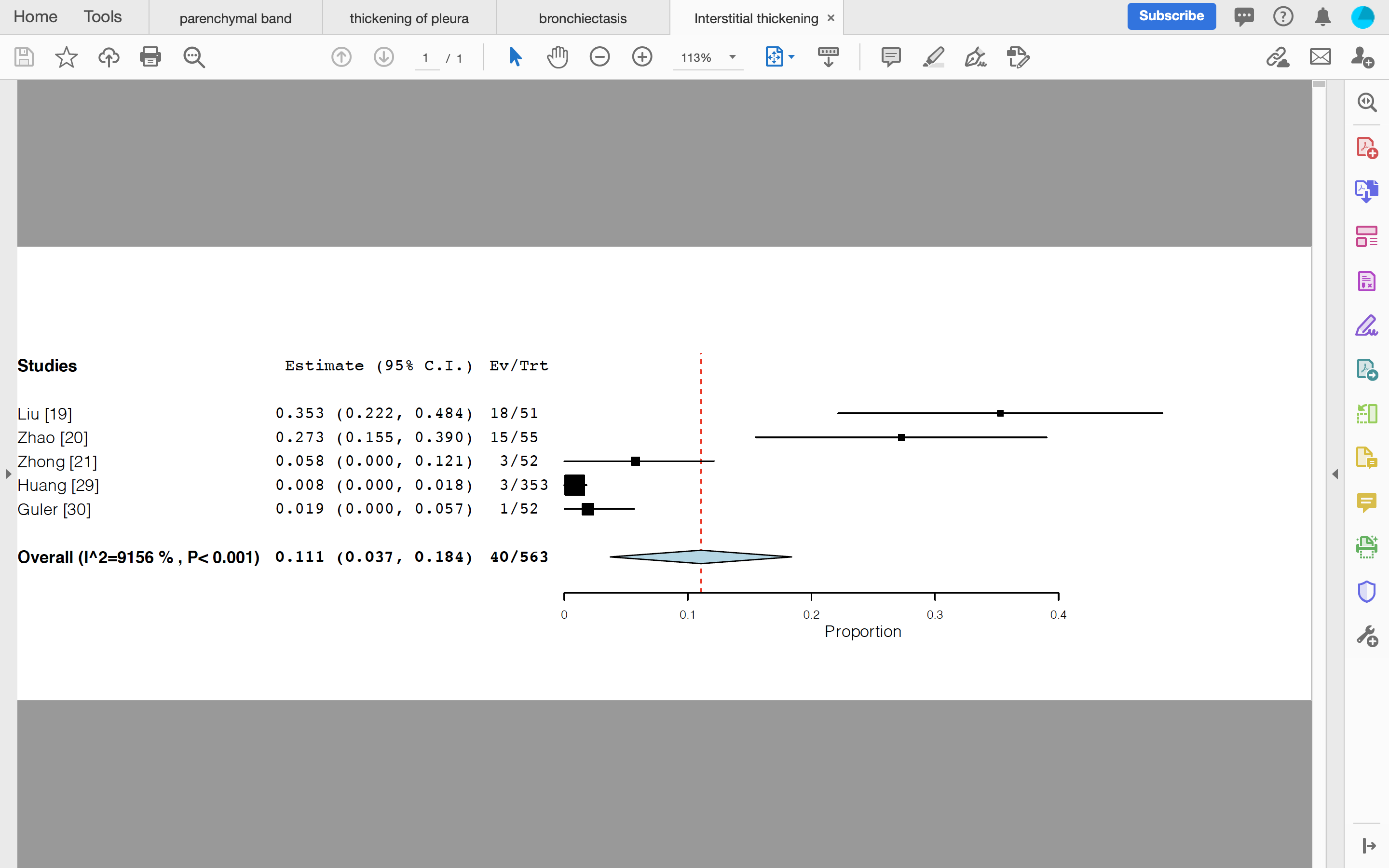


Supplemental Figure S1F


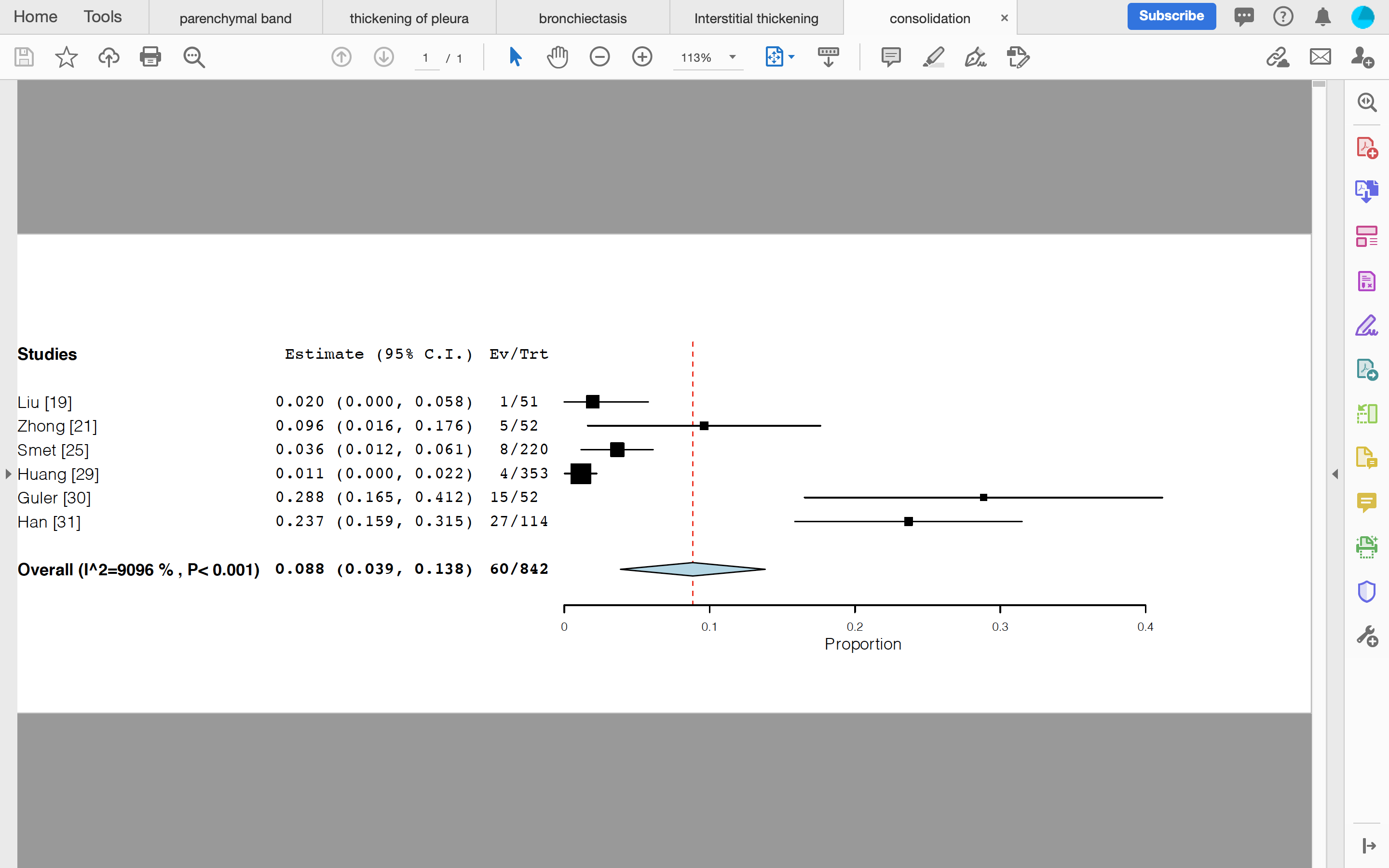


Supplemental Figure S1G


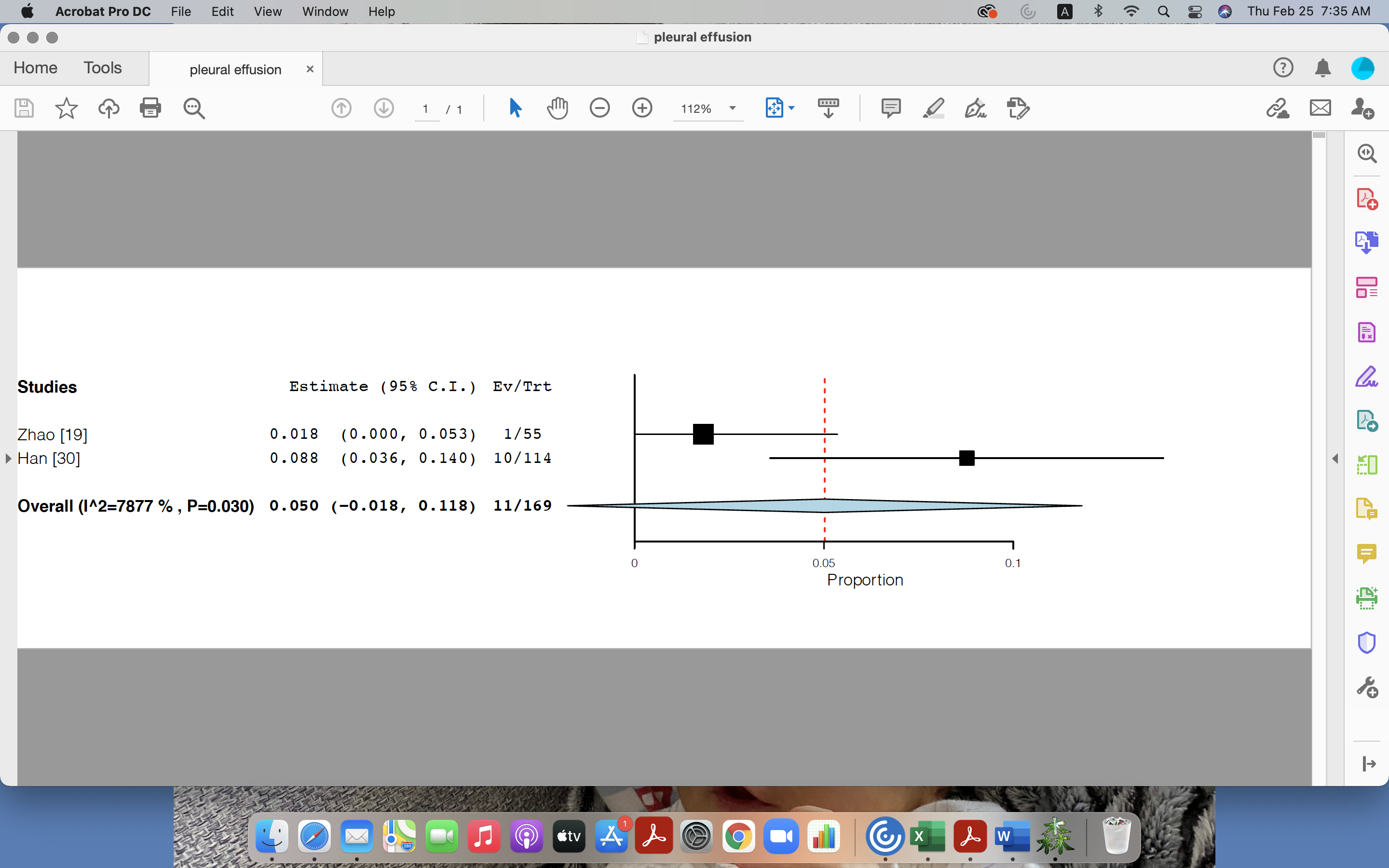


Supplemental Figure S2A


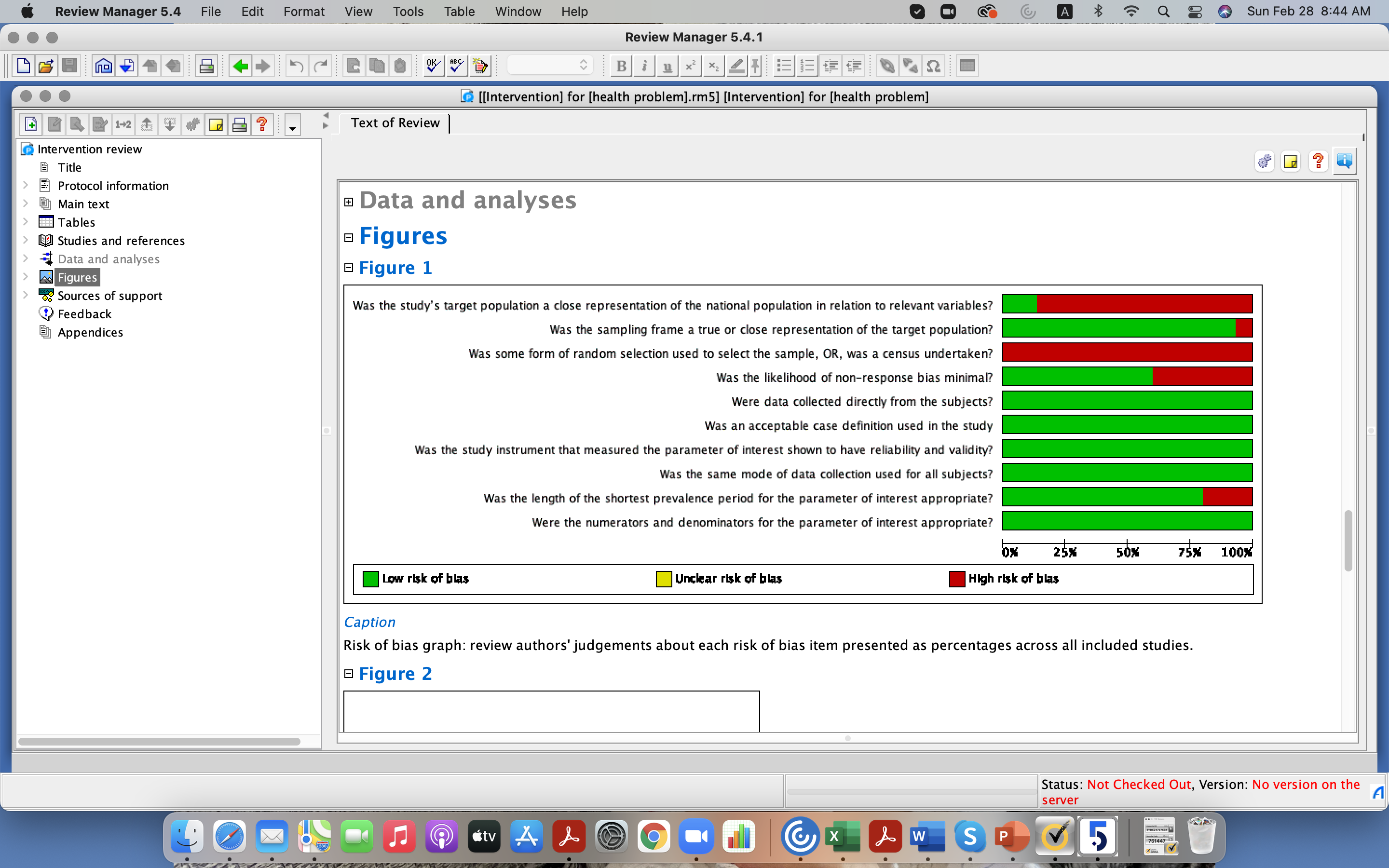


Supplemental Figure S2B


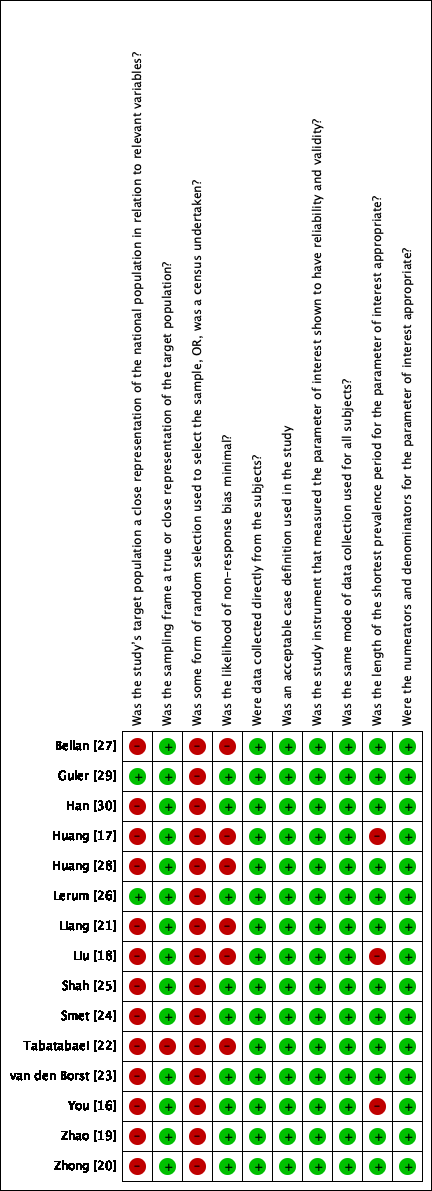


Supplemental Figure S3A

Egger’s test p value = 0.92

Supplemental Figure S3B

Egger’s test p value = 0.45

Supplemental Figure S3C

Egger’s test p value = 0.47

Supplemental Figure S3D

Egger’s test p value= 0.77

Supplemental Figure S3E

Egger’s test p value = 0.0372
